# First Report on the Prevalence and Subtype Distribution of *Blastocystis* sp. in Edible Marine Fish and Marine Mammals: A Large Scale-Study Conducted in Atlantic Northeast and on the Coasts of Northern France

**DOI:** 10.3390/microorganisms8030460

**Published:** 2020-03-24

**Authors:** Nausicaa Gantois, Angélique Lamot, Yuwalee Seesao, Colette Creusy, Luen-Luen Li, Sébastien Monchy, Sadia Benamrouz-Vanneste, Jacky Karpouzopoulos, Jean-Luc Bourgain, Célia Rault, Fabien Demaret, Martha Baydoun, Magali Chabé, Emilie Fréalle, Cécile-Marie Aliouat-Denis, Mélanie Gay, Gabriela Certad, Eric Viscogliosi

**Affiliations:** 1 Univ. Lille, CNRS, Inserm, CHU Lille, Institut Pasteur de Lille, U1019 – UMR 9017 – CIIL – Centre d’Infection et d’Immunité de Lille, F-59000 Lille, France; nausicaa.gantois@pasteur-lille.fr (N.G.); angelique.lamot@hotmail.com (A.L.); sadia.benamrouz@univ-catholille.fr (S.B.-V.); martha.e.b@hotmail.com (M.B.); magali.chabe@univ-lille.fr (M.C.); emilie.frealle@pasteur-lille.fr (E.F.); cecile.aliouat@univ-lille.fr (C.-M.A.-D.); gabriela.certad@pasteur-lille.fr (G.C.); 2 French Agency for Food, Environmental and Occupational Health and Safety (Anses), Laboratory for Food Safety, F-62200 Boulogne-sur-mer, France; seesao.yuwalee@gmail.com (Y.S.); luenlee@gmail.com (L.-L.L.); melanie.gay@anses.fr (M.G.); 3 Service d’Anatomie et de Cytologie Pathologiques, Groupement des Hôpitaux de l’Institut Catholique de Lille (GHICL), F-59000 Lille, France; creusy.colette@ghicl.net; 4 Univ. Littoral Côte d’Opale, CNRS, Univ. Lille, UMR 8187, LOG, Laboratoire d’Océanologie et de Géosciences, F-62930 Wimereux, France; Sebastien.Monchy@univ-littoral.fr; 5 Laboratoire Ecologie et Biodiversité, Faculté de Gestion Economie et Sciences, Institut Catholique de Lille, F-59000 Lille, France; 6 Coordination Mammalogique du Nord de la France, Groupe Mammifères Marins, F-62850 Alembon, France; jkarpouzopoulos@nordnet.fr (J.K.); jean-luc.bourgain@nausicaa.fr (J.-L.B.); celia.rault@yahoo.fr (C.R.); 7 Observatoire PELAGIS - UMS 3462, La Rochelle Université/CNRS, F-17000 La Rochelle, France; fdemaret@univ-lr.fr; 8 Délégation à la Recherche Clinique et à l’Innovation, Groupement des Hôpitaux de l’Institut Catholique de Lille, F-59000 Lille, France

**Keywords:** *Blastocystis* sp., edible marine fish, intestinal parasites, marine mammals, molecular epidemiology, real-time quantitative PCR, SSU rDNA sequence, subtyping, transmission, zoonosis

## Abstract

*Blastocystis* is frequently identified in humans and animal hosts and exhibits a large genetic diversity with the identification of 17 subtypes (STs). Despite its zoonotic potential, its prevalence and ST distribution in edible marine fish and marine mammals remain unknown. A large-scale survey was thus conducted by screening 345 fish caught in Atlantic Northeast and 29 marine mammals stranded on the coasts of northern France for the presence of the parasite using real-time Polymerase Chain Reaction PCR. The prevalence of the parasite was about 3.5% in marine fish. These animals were mostly colonized by poikilotherm-derived isolates not identified in humans and corresponding to potential new STs, indicating that fish are natural hosts of *Blastocystis*. Marine fishes are also carriers of human STs and represent a likely limited source of zoonotic transmission. 13.8% of the marine mammals tested were colonized and 6 different STs were identified including 3 potential new STs. The risk of zoonotic transmission through marine mammals is insignificant due to the lack of repeated contact with humans. The present survey represents the first data regarding the prevalence and ST distribution of *Blastocystis* in marine fish and marine mammals and provides new insights into its genetic diversity, host range and transmission.

## 1. Introduction

*Blastocystis* sp. is an anaerobic enteric protozoan frequently identified in humans and in a wide range of animal hosts, including various groups of mammals as well as birds, reptiles, amphibians and insects [1,2,3]. In the large majority of epidemiological studies conducted in recent years around the world and focused on intestinal parasites, *Blastocystis* sp. is by far the most common single-celled eukaryote found in human faeces. Indeed, its prevalence in humans reaches or can greatly exceed 50% in many geographical regions, particularly in developing countries where faecal peril represents a major risk in link with poor sanitary conditions, hygiene practices and low-quality drinking water [4,5,6]. The question of the pathogenic potential and clinical significance of *Blastocystis* sp. remains controversial so far because individuals colonized by the parasite are mostly asymptomatic [7,8,9]. However, several recent in vitro studies have clearly demonstrated the impact of the parasite on the intestinal epithelium of the host, underlining various virulence factors and mechanisms potentially involved in its pathogenesis [10]. Indeed, *Blastocystis* sp. infection was reported to be associated with non-specific intestinal disorders and possibly urticaria in numerous clinical case studies [11,12].

While human and animal isolates of the parasite do not exhibit significant morphological differences, an extensive genetic diversity has nevertheless been revealed between isolates of the genus *Blastocystis* through the comparison of the small subunit rDNA (SSU rDNA) sequences [8,9]. No less than 17 so-called subtypes (STs) have thus been successively identified so far among mammalian and avian isolates [13,14,15], and potential additional STs, so-called non-mammalian and avian STs (NMASTs), have also been proposed in amphibians, reptiles and insects [3,16]. Among the 17 mammalian and avian STs, 10 of them (ST1 to ST9 and ST12) were reported in humans, with varying prevalence [9,17,18]. A strong predominance of ST1 to ST4 was observed in the human population with more than 90% of all subtyped human isolates, which can be explained in large part by human-to-human transmission. The remaining STs, less frequently found in humans, colonize animal groups such as pigs for ST5, and birds for both ST6 and ST7, and these hosts represent potential reservoirs of zoonotic transmission [16]. Indeed, several evidences support the zoonotic potential of *Blastocystis* sp. such as the unusual high prevalence of the ST8, probably of animal origin, amongst zoo-keepers [19,20], as well as the sequence identity of ST5 isolates from pigs and piggery workers [21] and of ST6 isolates from poultry and slaughterhouse staff [22]. These data strongly suggest that repeated and direct contact between animals and their handlers could promote the transmission of *Blastocystis* sp. to humans.

Because of this risk of zoonotic transmission, the prevalence and ST distribution of *Blastocystis* sp. were reported in numerous surveys focused on various animal groups mainly housed in zoological gardens [15,16,20,23,24,25]. Despite the strong interest in identifying animal reservoirs of human infection, aquatic animals such as edible marine fish and marine mammals have received very little attention so far, and available data are still scarce. This can probably be explained by the difficulty in recovering intestinal samples from these animals for screening. To our knowledge, only a single study dating back to 1997 identified *Blastocystis* sp. in freshwater fish without molecular characterisation of the corresponding isolates [26] and no data are available regarding the eventual colonisation/infection of marine mammals by this parasite. In the particular case of fish, the consumption per year and per person has roughly doubled in 50 years and eating habits have also changed with an increase in the consumption of raw fish. Since fish species offered to the consumer are known to be frequently infested by various foodborne parasites [27,28], a real risk of transmission of *Blastocystis* sp. to humans exists through the simple handling and cleaning of these animals or by the consumption of undercooked fish.

The first aim of the present study was thus to determine the prevalence and ST distribution of *Blastocystis* sp. by screening the four most sold and consumed edible marine fish in the region Hauts-de-France that were caught in Atlantic Northeast, and marine mammals stranded on the coasts of northern France. The second goal was to evaluate the potential risk of zoonotic transmission of the parasite through the comparative analysis of the ST distribution and sequences of isolates identified in edible marine fish and marine mammals with those reported in the human population.

## 2. Materials and Methods

### 2.1. Ethics Approval

No approval from the institutional animal care and use or the ethics committee was necessary in regard to the French law as no experiment involving living fish was performed. To obtain marine mammalian samples, an agreement (2019-R-03) was signed between the PELAGIS Observatory (Observatory for Sea Mammals and Seabirds Conservation, UMS 3462, La Rochelle, France), the local correspondents of the National Stranding Network (NSN) (http://www.observatoire-pelagis.cnrs.fr/observatoire/Suivi-des-echouages-37/) and the Anses at Boulogne-sur-Mer. A sampling request was completed each year of the study and submitted to the NSN evaluation committee for renewal.

### 2.2. Fish Sampling

**Figure 1:**
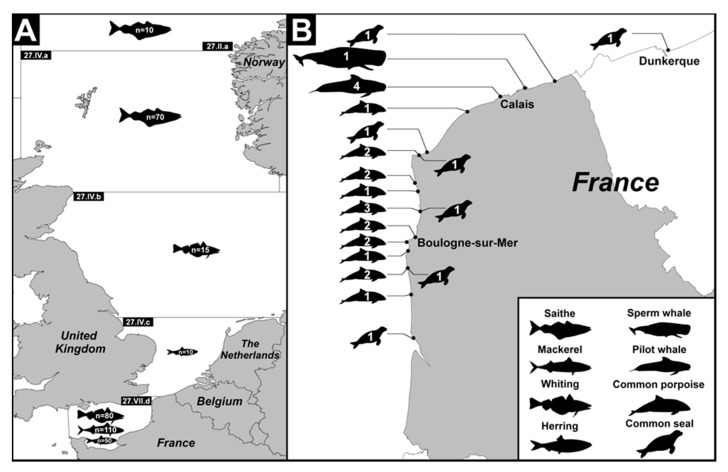
Detailed boundaries of the fishing sub-areas within the Atlantic Northeast zone 27 (**A**) and the geographical location of marine mammal stranding sites (**B**). The number of fishes collected for the selected species in each sub-area is indicated as well as the number of marine mammals and the corresponding species for each stranded site on the coasts of northern France.

In the present study, fish sampling was carried out at Boulogne-sur-Mer (Coordinates: 50° 43’ N-1° 37’ E), the first French fishing port. The 4 most sold and consumed marine fish species in the region Hauts-de-France, i.e., *Clupea harengus* (herring) belonging to the order Clupeiformes, *Scomber scombrus* (mackerel) belonging to the order Perciformes and *Merlangius merlangus* (whiting) and *Pollachius virens* (saithe) both belonging to the order Gadiformes, were selected. A sampling schedule was established to collect samples of each selected species at different times of the fishing season. Overall, 345 fishes were obtained from wholesalers over a period of 2 consecutive years and distributed into 6 to 7 batches of twenty to thirty fresh un-gutted marine fishes for each selected species (Table 1 and Figure 1A). For each batch, the fishing date and fishing sub-area within Atlantic Northeast zone 27 as determined by the International Council for the Exploration of the Sea (ICES; https://www.ices.dk/) were recorded (Eastern English Channel, Southern North Sea, Central North Sea, Northern North Sea or Norwegian Sea). With the exception of 3 batches analysed entirely, the first 10 to 20 fish from each batch (Table 1) were screened for *Blastocystis* sp. For each fish, a part of the intestinal content (IC) was collected after dissection and stored in Stool Transport and Recovery (S.T.A.R.) STAR Buffer (Roche Life Science, Meyran, France) at 4 °C. Scrapings of a part of the intestinal (SIE) and stomach (SGE) epithelia were also performed and the cells were preserved in RNA later Stabilization Solution (Invitrogen, Groningen, the Netherlands) at −20 °C before use. In addition, sections of the stomach and bowel were fixed in 10% buffered formalin for histological analysis.

### 2.3. Marine Mammal Sampling

This part of the study was conducted in close collaboration with the PELAGIS Observatory, which coordinates the action of the NSN to collect data on marine mammals stranded along the entire French coastline. As soon as a marine mammal stranding was reported on the coasts of Hauts-de-France (Figure 1B), local correspondents of the NSN (Coordination Mammalogique du Nord de la France) were sent on site to collect various biological samples and complete a standardised questionnaire summarizing information of interest, such as species, sex, size, estimated weight and conservation status (fresh, very fresh or putrefied) of the animal, together with date and location of stranding and potential causes of death. Study sampling was inherently strongly dependent on stranding conditions and carried out only on exploitable individuals. Stranded animals were immediately dissected by NSN members on site and the entire digestive tract or part of it was at once transported to the Laboratory for Food Safety of Anses at Boulogne-sur-Mer, France. After opening the digestive tract, a part of the intestinal content was collected and stored in STAR Buffer at 4 °C, as for fishes. In parallel, scrapings of different parts of the gastric epithelium including forestomach (SGE-FO), fundic stomach (SGE-FU) and pyloric stomach (SGE-PY), and of the intestinal epithelium, were performed and stored in RNA later Stabilization Solution at −20°C. However, marine mammals were not all found in excellent conditions of preservation after stranding and, consequently, all the different samples were not necessarily collected for each animal tested. In the end, a total of 76 marine mammal samples were collected and analysed from 29 cetaceans and pinnipeds (Table 2). No histological analysis was performed due to insufficient animal tissue preservation.

### 2.4. DNA Extraction

For the detection of *Blastocystis* sp., total genomic DNA was extracted directly from approximately 250 mg of all intestinal content samples of both edible marine fish and marine mammals using the QIAamp DNA Stool Mini Kit (Qiagen GmbH, Hilden, Germany) following the instructions provided in the kit. DNA was eluted in 100 μL of AE buffer (Qiagen GmbH, Hilden, Germany). For the scrapings of the gastric and intestinal epithelia, total DNA was extracted using the NucleoSpin^®^ tissue kit (Macherey-Nagel, Düren, Germany) according to the manufacturer’s recommended procedures. DNA extraction negative controls were included. All DNAs obtained were stored at −20°C until being analysed.

### 2.5. Amplification of the SSU rDNA Gene and Molecular Subtyping of Blastocystis sp. Isolates

For each sample tested, 2 μL of extracted DNA was subjected to a real time PCR (qPCR) assay using the *Blastocystis*-specific primers BL18SPPF1 (5′-AGTAGTCATACGCTCGTCTCAAA-3′) and BL18SR2PP (5′-TCTTCGTTACCCGTTACTGC-3′) targeting the SSU rDNA gene as previously described [29]. DNA extraction controls were subsequently used in qPCR assays and both positive (DNA obtained from *Blastocystis* sp. ST7 and ST4 axenic cultures maintained in the laboratory) and negative (DNA matrix replaced by water) qPCR controls were included. The positive qPCR products were purified and directly sequenced on both strands in a sequencing facility (Genoscreen, Lille, France). In case of a single marine mammal sample (CET-09 SIE), direct sequencing of the qPCR product generated a mixed signal that could reflect a mixed infection, i.e., infection by at least two *Blastocystis* STs. This sample was thus re-analysed by non-qPCR using the same primer pair as for qPCR. End-point PCR was performed in 50 µl according to standard conditions for Platinum *Taq* High-Fidelity DNA polymerase (Invitrogen, Groningen, the Netherlands). Positive and negative controls were included. Briefly, after a denaturation step at 94 °C for 5 min followed by 40 cycles of amplification (30 s at 94 °C, 35 s at 60 °C, 50 s at 68 °C) and a final extension step at 68 °C for 2 min using a Bioer LifeECO apparatus (Binjiang District, China), the end-point PCR product was separated by agarose gel electrophoresis and the band of the expected size (approximately 320 bp) was purified using the Wizard SV Gel and PCR clean-up system (Promega, Madison, WI, USA). The purified PCR product was cloned in the T-vector, pCR 2.1-TOPO (Invitrogen, Groningen, the Netherlands), and amplified in *Escherichia coli* One Shot TOP10 competent cells. Five positive clones containing inserts were arbitrarily selected. Minipreparations of the corresponding plasmid DNAs containing inserts were done using the NucleoSpin Plasmid kit (Macherey-Nagel, Düren, Germany) and sequenced on both strands. All SSU rDNA sequences obtained in this study were deposited in GenBank under accession numbers MN439925 to MN439951. These sequences were compared with all *Blastocystis* sp. homologous sequences available from the National Centre for Biotechnology Information (NCBI) using the nucleotide Basic Local Alignment Search Tool (BLAST) program.

Considering the large number of edible marine fishes analysed in the present study and considering that *Blastocystis* sp. is recognized as an extracellular parasite, the strategy for research and identification of this microorganism in the different samples was as follows: for all fishes, the IC sample was first tested by qPCR assay for the presence of the parasite, as detailed previously. If the qPCR assay was negative, the fish was considered as not colonized by the parasite. If the qPCR assay was positive, both SIE and SGE samples were tested by qPCR followed by histological analysis (see below) in case of positive results.

### 2.6. Phylogenetic Analysis of Blastocystis sp. Isolates

Ten SSU rDNA sequences obtained in the present study from *Blastocystis* sp. isolates identified in edible marine fish and three sequences obtained from marine mammal isolates exhibited low similarity (≤92%) with homologous sequences of known STs available in databases. To clarify their identification through a large-scale phylogenetic analysis, these 13 sequences were added to a dataset, including (i) 33 homologous sequences of *Blastocystis* sp. isolates representative of ST1 to ST10 and ST13 to ST17 (ST11 and ST12 SSU rDNA sequences are not yet available for the amplified domain) [20], (ii) 24 sequences representing 7 potential NMASTs according to recent phylogenetic analyses [3,16] and 6 sequences corresponding to animal isolates with undefined classification, 5 of them recently assigned as un-typable [16]. The SSU rDNA sequences were aligned using the BioEdit v7.2.5 package (www.mbio.ncsu.edu/BioEdit/bioedit.html). All positions containing gaps were eliminated and the phylogenetic inference was restricted to 254 sites that could be unambiguously aligned. Phylogenetic analyses were performed using maximum likelihood methods implemented in MEGA6 [30] and MrBayes. The maximum likelihood analysis was based on the General Time Reversible model [31], and initial trees for the heuristic search were obtained by applying the Neighbour-Joining method to a matrix of pairwise distances estimated using the Maximum Composite Likelihood approach. A discrete Gamma distribution was used to model evolutionary rate differences among sites (8 categories (+G, parameter = 0.3435)). The rate variation model allowed for some sites to be evolutionarily invariable ((+I), 0.0000% sites). Bootstrap proportions (BPs) were obtained from 1000 pseudo-replicates. Bayesian Posterior Probabilities (BPPs) were calculated from 1000 replicates using MrBayes 3.2.6 with the maximum likelihood method for 10 million Markov Chain Monte Carlo generations.

### 2.7. Histological Analysis

Paraffin-embedded tissues of digestive organs previously fixed in formalin were sectioned to a thickness of 5 μm to be processed using standard staining techniques (Hematoxylin and eosin). The sections were observed using a Leica DMRB microscope equipped with a Leica digital camera connected to an Imaging Research MCID analysis system (MCID Software, Cambridge, United Kingdom).

### 2.8. Statistical Analysis

Fisher’s exact test was used to test the relationship between different categorical variables. A logistic regression model was used to calculate odds ratios (OR) with *Blastocystis* sp. presence as the main outcome. The general significance level was set at a *p*-value below 0.05. Statistical analyses were performed using the VassarStats software (http://vassarstats.net/).

## 3. Results and Discussion

### 3.1. Prevalence of Blastocystis sp. in Edible Marine Fish and Marine Mammal Samples

In total, 345 fishes were collected and distributed as follows: 60 herring, 95 whiting, 80 saithe and 110 mackerel (Table 1). After molecular analysis of intestinal contents by qPCR, the *Blastocystis* sp. prevalence was 3.5% (12/345) but with variations between fish species. Indeed, this prevalence reached 8.3% (5/60) in herring but was only 2.1% (2/95) in whiting, 2.5% (2/80) in saithe and 2.7% (3/110) in mackerel (Table 1 and Table 3). The risk of detection of *Blastocystis* sp. in herring was four-fold higher compared with the other species (OR: 4, Confidence Interval (CI)CI: 1.10–11.79, *p* = 0.04). While this observation has to be confirmed in future surveys, our data suggest that some fish species would be more prone to carry *Blastocystis* sp. Interestingly, a previous study screening the same fish specimens in the aim to identify the intestinal protozoa *Cryptosporidium* spp. showed that the prevalence was the highest in saithe for unknown reasons [28]. For comparison, the only study available in the literature on the presence of *Blastocystis* sp. in fish indicated a prevalence of 11% from a limited size group of 18 tropical and subtropical freshwater fish, and after isolation of the parasite by culture [26].

The prevalence values observed by fishing area also showed variations since 4.2% of fishes (10 out of a total of 240) caught in the Eastern English Channel (fishing area 27.VII.d) and 2.9% (2/70) in the Northern North Sea (fishing area 27.VII.a) were colonized by the parasite. On the other hand, specimens from either the Central North Sea or the Norwegian Sea were found to be negative (0/15 and 0/10, respectively) (Table 3). This variation could likely be due to the differences in the prevalence of the parasite between fish species within each fishing area. Indeed, herring had the highest prevalence of *Blastocystis* sp. and was mostly caught in the Eastern English Channel, probably increasing the overall prevalence of the parasite in this particular fishing area. However, this difference in the prevalence of the parasite between fishing areas was not statistically significant (*p* = 0.24).

Strikingly, almost all fishes infected by *Blastocystis* sp. (11/12) were caught in autumn and winter (Table 1 and Table 3), with a 14-times higher risk of parasite presence in this group when compared to the fishes collected in spring–summer (OR: 14, CI: 1.73–106, *p* < 0.001). Even if no explanation can yet be provided, a seasonal effect could be highlighted since no fish caught from April to August were positive for *Blastocystis* sp. Seasonality of *Cryptosporidium* spp. distribution in fish has also been described but with maximal prevalence occurring in spring and summer [26].

Among the 12 fishes positive for *Blastocystis* sp. in their IC, the parasite was also identified in the SGE of one herring (CH-II-8) and one saithe (PV-IV-3), as well as in both SIE and SGE of the herrings CH-II-1 and CH-II-7 (Table 3), implying that *Blastocystis* sp. would be able to colonize different parts of the gastrointestinal tract of fish.

In total, 29 stranded marine mammals were also screened in the present study, including 22 cetaceans (common porpoise, long-finned pilot whale and sperm whale) and 7 pinnipeds (common seal) (Table 2). The molecular analysis by qPCR allowed the identification of *Blastocystis* sp. in 4 of these animals, which resulted in a prevalence of 13.8%. As shown in Table 4, the positive samples corresponded to 3 cetaceans (common porpoises CET-08 and CET-09 and sperm whale CET-15) and 1 pinniped (common seal PIN-06). Moreover, among the 76 marine mammal samples analysed, 7 of them (9.2%) were shown to be positive for *Blastocystis* sp. For the common porpoise CET-08 and the sperm whale CET-15, *Blastocystis* sp. was only identified in SIE, while for the common seal PIN-06, the parasite was found in both IC and SIE. In the case of the common porpoise CET-09, *Blastocystis* sp. was identified in various sites of the digestive tract (SIE, SGE-FU and SGE-FO), suggesting that the parasite was able to colonize different parts of the intestinal tract, as described above for fishes. To the best of our knowledge, this study presents the first report of *Blastocystis* sp. prevalence in marine mammals and should be extended to other species of interest in order to assess the real impact of this parasite in this group of animals.

### 3.2. ST Distribution of Blastocystis sp. in Edible Marine Fish

As mentioned above, only one study investigated the presence of *Blastocystis* sp. in fish and identified 2 animals positive for the parasite [26]. Briefly, only one of these two isolates was characterised, but not through molecular tools. Indeed, by using antisera prepared against *Blastocystis* sp. human strains, this isolate of fish was identified as belonging to the so-called serogroup II, together with some other mammalian and avian isolates. However, this classification in serogroups was not correlated to any ST in the standardised terminology of *Blastocystis* [14] and consequently, no data on parasite STs colonizing fish was available.

In the context of several recent large-scale epidemiological studies including human and/or animal cohorts [16,32], the partial sequence of the SSU rDNA gene amplified by the qPCR assay used in the present study has already been shown to provide sufficient information for differentiating STs of *Blastocystis* sp. by direct sequencing of amplicons. Excluding primers, the DNA fragments sequenced herein from edible marine fish samples were 288 to 301 bp in size, depending on ST. The STs were identified by determining the exact match or closest similarity against all known *Blastocystis* sp. STs according to the most recent classifications of the parasite [15,16]. First, this comparison allowed us to confirm that the analysed sequence corresponded to *Blastocystis* sp. and then to determine the corresponding ST based on the percentage of identity obtained by BLAST. When the level of sequence similarity reaches or exceeds 95% with a known ST, the amplicon is considered to belong to the considered ST [9].

Four out of 12 *Blastocystis* sp.-positive fishes (CH-II-1, CH-II-7, CH-II-8 and PV-IV-3) were shown to be colonized by different isolates of the parasite, resulting in the subtyping of a total of 18 isolates from this animal group (Table 3). These isolates showed 85% to 100% identity with homologous sequences of *Blastocystis* sp. available in databases. Among these isolates, five of them identified in herring and saithe exhibited 100% sequence identity with each other and were classified as ST8. Indeed, the SSU rDNA sequence of these isolates exhibited 100% identity with those of several ST8 isolates identified from humans and animal hosts in different countries [16,33,34]. As reported in a recent epidemiological survey conducted in zoos and compiling available data from the literature [16], ST8 has been almost exclusively identified in non-human primates, to a lesser extent in marsupials and episodically in human beings in relation to a likely zoonotic transmission [16]. Indeed, while the ST8 has sporadically been isolated from humans in general, it was frequently found in zookeepers in contact with non-human primates, suggesting zoonotic spread from primates to primate handlers [19]. Because of the distribution of ST8 isolates in the animal kingdom, it seems highly unlikely that fish represent a natural host of this ST despite having been identified in two fish species caught in the Eastern English Channel and the Northern North Sea, respectively. Consequently, the presence of ST8 in fish samples might be related to local water contamination by animal or human wastes. In a recent study conducted in central/southern Sweden [35], ST8 was surprisingly identified in almost 40% of the untreated wastewater samples tested. However, as discussed by the authors, the host, whether human or animal, contributing to the sewage influent remains as yet unidentified. Interestingly, herring, when mature, is known to spawn along the coast or in shallow waters, which may explain its colonisation by ST8 while staying in contaminated water areas. More globally, this behaviour may also be correlated with the relatively high level of infection by *Blastocystis* sp. described in herring in comparison to other fish species.

Two additional isolates from whiting and mackerel, both caught in the Eastern English Channel and presenting identical SSU rDNA gene sequences, were identified as belonging to ST2. These ST2 sequences exhibited 100% identity with those of various isolates found in humans and different animal groups. Indeed, ST2 is one of the four most predominant STs in the human population, particularly in Europe [8,9,29,32], and was commonly identified in numerous major animal groups including non-human primates, carnivores, artiodactyls, rodents and birds [16]. Therefore, the low host specificity of *Blastocystis* sp. ST2 coupled to its low prevalence in edible marine fishes suggest that these animals do not seem to be natural hosts of ST2, as proposed above for ST8. As a result, the hypothesis of a transient ST2 infection in marine fish through seawater contaminated by animal or human faecal material is highly probable. A similar hypothesis was also proposed for an isolate identified in a herring (CH-II-8) caught in the Eastern English Channel and belonging to ST7. Indeed, the SSU rDNA sequence of this isolate showed 98% identity with homologous sequences from human and avian isolates. This ST together with ST6 are considered as avian STs because of their predominance in birds and were shown to be transmissible to humans by a zoonotic route [9,16,17,22,36]. Consequently, the single fish presenting *Blastocystis* sp. ST7 could have been colonized by the parasite as a result of contact with seawater likely contaminated with bird faeces.

**Figure 2:**
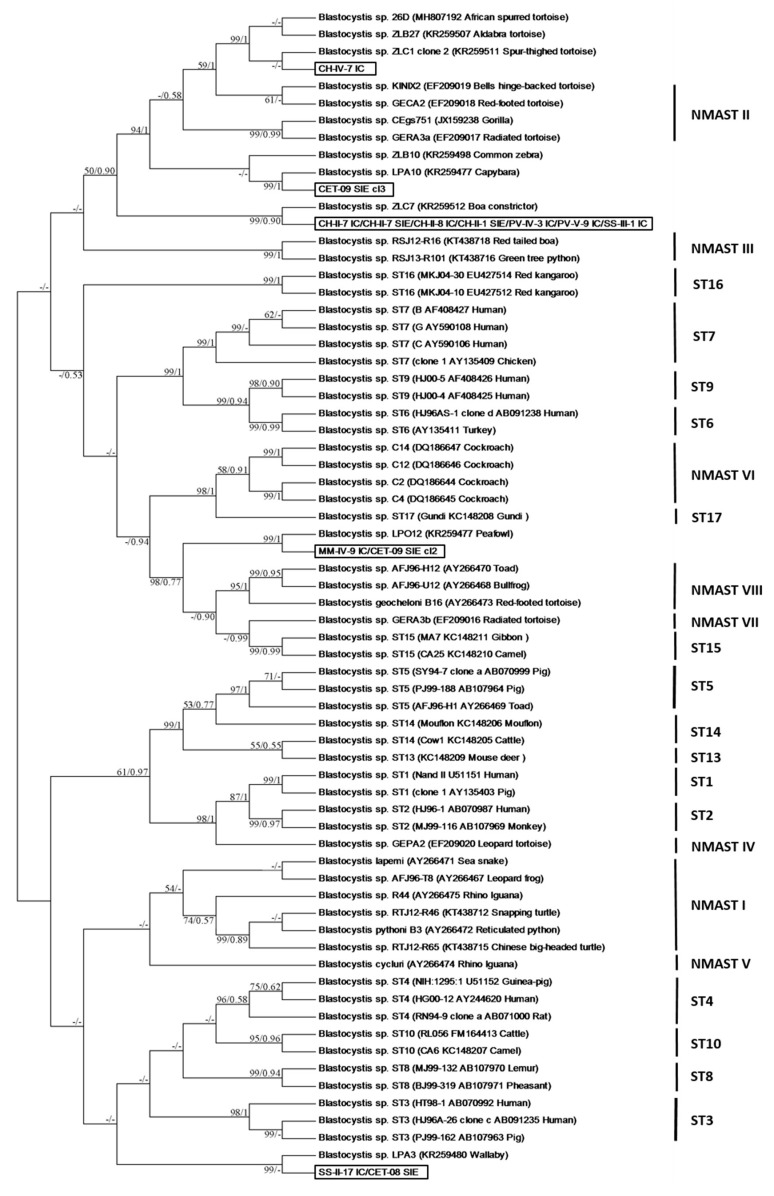
Unrooted maximum likelihood cladogram based on SSU rDNA sequences depicting relationships between *Blastocystis* sp. isolates. Only the tree with the highest log likelihood (−2762.8734) is shown. For each sequence extracted from the databases, its accession number, the potential name of the corresponding isolate and its host are indicated. Numbers near the individual nodes correspond to BP (left of the slash) and BPP (right of the slash) given by the two different tree reconstruction methods (Maximum Likelihood/MrBAYES). The asterisks designate nodes with BPs or BPPs below 50% or 0.5, respectively. The sequences obtained in the present study are shown in bold and in boxes.

The remaining 10 SSU rDNA sequences obtained from edible marine fish isolates exhibited 85% to 92% identity with homologous sequences available from isolates of known STs, thus preventing direct subtyping. Consequently, to clarify the origin and relationships of these isolates with those of known STs, their sequences were included in a large phylogenetic analysis including 57 homologous sequences from representatives of both mammalian and avian STs and NMASTs together with 6 unidentified sequences from animal isolates [16]. In our unrooted maximum likelihood tree (Figure 2), all mammalian and avian STs formed monophyletic groups strongly supported by BP and BPP values, with the exception of ST14 that represented a paraphyletic clade. Despite its short length, the compared domain of the SSU rDNA gene could be considered as a valuable phylogenetic marker since the topology of the present tree was extremely similar to that of the tree based on full-length sequences of the same gene [3]. Moreover, the eight NMASTs previously proposed [16], called NMAST I to NMAST VIII, and which represented either reptilian, amphibian/reptilian or insects clusters, were all resolved in the present phylogenetic analysis, with the exception of the paraphyletic clade NMAST II.

Strikingly, 7 out of the 10 sequences of fish isolates included in the present analysis (CH-II-7 IC, CH-II-7 IE, CH-II-8 IC, CH-II-1 SIE, PV-IV-3 IC, PV-V-9 IC, SS-III-1 IC) exhibited 100% identity with each other and corresponded to isolates identified in three different fish species (herring, saithe and mackerel) caught in two different geographical areas (Eastern English Channel and Northern North Sea). These sequences showed only 92% identity with those of ST10 isolates found in Artiodactyla and mainly bovid, which are considered as natural hosts of this ST [16,22,24,37,38], but were identical to that of the untypable isolate ZLC7 identified in a reptile (*Boa constrictor*) housed in the zoo of Lille in France [16]. As shown in our phylogenetic tree, these 7 sequences emerged at the basis of a large group including, more specifically, the reptilian cluster NMAST II with BP and BPP of 50% and 0.90 respectively, and formed a sister group with reptilian isolates belonging to NMAST III. In view of its separate emergence and large evolutionary distances with neighbouring clusters, this lineage composed of the sequences of these 7 fish isolates and that of the reptilian isolate ZLC7 was likely representative of a new ST. This hypothesis remains to be confirmed by further phylogenetic analyses based on complete SSU rDNA sequences for the corresponding isolates. In fact, this new potential lineage could represent a ST adapted to poikilothermic animals and especially fish in view of its significant prevalence in this group of animals and its identification in various fish species of different geographical origins.

The SSU rDNA sequence corresponding to another isolate identified in herring (CH-IV-7 IC) showed 91% identity with those of NMAST II isolates and exhibited 99% to 100% identity with those of the untypable isolates ZLB27 and ZLC1 and the isolate 26D, all 3 found in tortoise species. Consequently, the CH-IV-7 IC sequence and those of the 3 latter isolates formed a strongly supported clade (BP and BPP of 99% and 1, respectively) as a sister group of the NMAST II lineage. These data suggest that these 4 isolates could form another potential new ST that would also be specific of poikilothermic animals since it was identified in both fish and tortoises.

The last 2 isolates identified in edible marine fishes, namely MM-IV-9 IC from whiting and SS-II-17 IC from mackerel, exhibited a very distant emergence from those of other fish isolates in our phylogenetic tree. Indeed, the corresponding SSU rDNA sequences of these two isolates exhibited 85% and 91% identity with NMAST VII and ST8 isolates, respectively. However, these isolates also showed 100% sequence identity with those of the un-ypable LPO12 and LPA3 isolates found in peafowl and wallaby, respectively [16]. As shown in our SSU rDNA-based phylogeny, the MM-IV-9 IC and LPO12 isolates were strongly assigned (BP and BPP of 98% and 0.77, respectively) at the basis of a large group including amphibian/reptilian isolates of the NMAST VII and NMAST VIII and non-human primate isolates belonging to ST15. Regarding the isolates SS-II-17 IC and LPA3, both formed a lineage with unresolved emergence in basal position of a cluster grouping together ST3, ST4, ST8 and ST10 isolates from homoeothermic animals. In view of their respective emergence and limited composition in terms of isolates originating from different hosts, it still remains unclear at first whether these two last lineages corresponded to potential new STs and secondly, whether both STs could eventually represent fish-adapted STs or whether their presence in fish was related to contaminated seawater by unidentified animal waste.

### 3.3. ST Distribution of Blastocystis sp. in Marine Mammals

This study also provides the first data on the distribution of *Blastocystis* sp. STs in marine mammals. Excluding primers, the DNA fragments sequenced in the present study from marine mammal samples found positive by qPCR were 277 to 301 bp in size. Among the 7 marine mammal samples identified as sequence-positive for the parasite (Table 4), 6 presented single infections by *Blastocystis* sp. The latter sample (CET-09 SIE from common porpoise) exhibited a mixed infection according to the sequence trace requiring cloning of the non-qPCR product. After cloning followed by sequencing of 5 positive clones, 3 different STs were identified from this sample. In total, 9 isolates were subtyped and presented 82% to 100% identity with homologous sequences of known STs available in databases (Table 4). Despite the limited number of samples analysed, a wide diversity of STs have been found in marine mammals since 6 different potential STs were identified. In addition, in a common porpoise (CET-09), no less than 5 potential STs have been found colonizing different parts of its digestive tract. In particular, the scraping of its intestinal epithelium was colonized by at least 3 different STs of *Blastocystis* sp., suggesting successive infections through probably multiple sources of contamination.

Among these isolates, 6 of them identified in either common porpoise, sperm whale or common seal were easily subtyped since the corresponding SSU rDNA sequences exhibited 99% to 100% identity with those of either ST2 (CET-09 SGE-FU and CET-15 SIE), ST3 (CET-09 SGE-FO) or ST4 (CET-09 SIE, PIN-06 IC and PIN-06 SIE) isolates available in databases. These 3 STs are commonly found in humans and numerous animal groups, including terrestrial mammals such as non-human primates, Carnivora and Artiodactyla [9,15,16,17]. Hence, our data showed that these 3 STs could colonize both terrestrial and marine mammalian groups. Further studies to increase the number of samples and species screened have to be conducted with the aim of confirming that marine mammals represent natural hosts of *Blastocystis* sp. Indeed, the transient infection by the parasite through exposure to human or animal waste cannot be ruled out.

The sequences of the other 3 isolates identified in marine mammals (CET-08 SIE, CET-09 SIE clone 2 and CET-09 SIE clone 3 from common porpoise) presented 82% to 91% identity with those of isolates of known STs and were therefore included in our phylogenetic analysis (Figure 2). The sequence of the first isolate CET-08 SIE exhibited 91% identity with isolates belonging to ST8 but 100% identity with those of the mackerel isolate SS-II-17 IC described above and of the untypable isolate LPA3 from wallaby. The sequence of the second isolate CET-09 SIE clone 2 showed only 85% identity with that of a NMAST VII isolate from tortoise but was identical to that of the fish isolate MM-IV-9 IC from whiting and exhibited 99% identity with that of the untypable isolate LPO12 from peafowl. Consequently, the isolates CET-08-SIE and CET-09-SIE clone 2 were both representatives of strongly supported lineages composed of only 3 isolates from fish, marine mammal and either bird or marsupial. The fact of identifying in each of these two lineages an isolate common to a fish and a marine mammal could suggest a transmission of the parasite to the marine mammal through the consumption of infected fish. However, the origin and potential host-specificity of these two lineages remain uncertain at this time and have to be further elucidated.

The sequence of the isolate CET-09 SIE clone 3 was very divergent compared to those of known STs. Indeed, it exhibited only 82% sequence identity with that of the isolate GECA2 from tortoise representative of NMAST II. On the other hand, this sequence showed 99% identity with that of the untypable isolate LPA10 from capybara. Consequently, isolates CET-09-SIE clone 3 and LPA10 grouped together with BP and BPP of 100% and 1 respectively, in our phylogenetic tree and formed with the untypable isolate ZLB10 from common zebra, a cluster only supported by BPP and including mammalian hosts. However, this cluster was included in a well-supported clade (BP and BPP of 52% and 0.87) composed of numerous isolates mostly from reptiles such as those belonging to NMAST II, together with those of fishes according to our present data. Moreover, this large clade also formed a sister group with the reptilian NMAST III sequences but with low BP and BPP supports. Therefore, it becomes unclear whether this group including the isolate CET-09 SIE clone 3 represents a cluster of mammalian isolates or whether it reflects a transient colonisation of mammals by isolates of poikilothermic animals.

### 3.4. Colonisation and Circulation of Blastocystis sp. in Edible Marine Fish and Marine Mammals and Risk of Zoonotic Transmission

**Figure 3:**
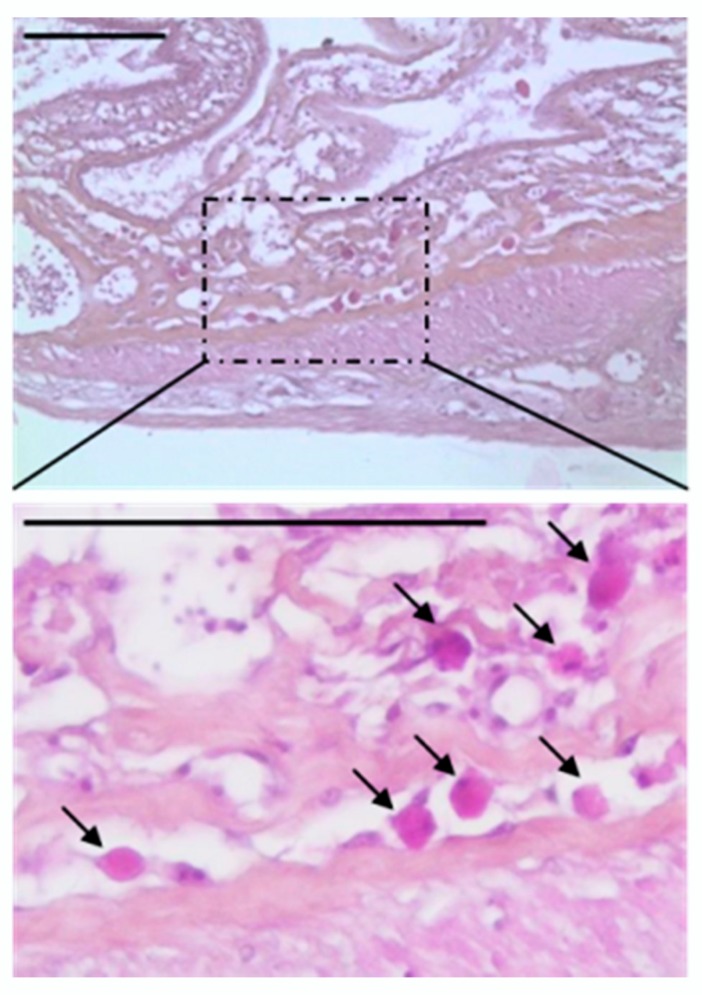
Hematoxylin and eosin-stained section of the bowel of herring CH-II-7. The presence in large numbers of round bodies (arrows) is suggestive of *Blastocystis* sp. vacuolar forms located in the tissue. Scale bar = 100 µm.

With regard to the significant prevalence of *Blastocystis* sp. in edible marine fish and marine mammals, the question of the colonisation *versus* infection of these animals by the parasite was clearly raised, hence the interest in the analysis of digestive histological sections of infected individuals. Unfortunately, the presence of *Blastocystis* sp. could not be detected in the stomach and/or intestine of all infected animals due eventually to lysis of tissues and small parasite size. However, vacuolar-like forms of the parasite were clearly identified at least in sections of the intestine of one herring (CH-II-7) (Figure 3). Interestingly, through the analysis of these bowel sections, a large number of parasites were observed in the intestinal lumen of fish likely suggesting the multiplication of the parasite and a true infection of the fish by *Blastocystis* sp. rather than a carriage. In addition, some of the parasites were shown to adhere to the intestinal epithelium of the host and others appear to be located inside the tissue. This observation was particularly interesting since an isolate suggested to be representative of a new ST likely colonizing poikilothermic animals was identified in both the IC and SIE of the herring CH-II-7. Therefore, this observation that has to be confirmed in further analyses and may possibly be extended to other fishes infected with the same isolate.

Two main hypotheses could be proposed regarding transmission of *Blastocystis* sp. to fish. The first and probably most likely was that of fish-to-fish transmission of the parasite occurring during cohabitation in dense groupings, as suggested, for instance, in social and gregarious gadid fish [39]. A second hypothesis would be the transmission of the parasite through the food chain and principally the consumption of smaller fish and other small-sized aquatic animals potentially carrying *Blastocystis* sp. since the 4 fish species included in our survey were all predatory. Similar mode of transmission of the parasite could be proposed in marine mammals that are known to ingest massive quantities of food from their aquatic environment. This consumption of aquatic animals during the lifetime of the edible marine fishes and marine mammals could promote parasite infection and its accumulation in these hosts. The identification of various STs in the same animal as described for several individuals in the present study was probably due to this reason. Focusing more carefully on certain STs identified in fishes and also found in terrestrial mammals, an exposure to contaminated water with animal or human wastes could logically be suggested. Although fishes analysed in the present study were sampled offshore and contamination through coastal water would appear to be less significant, this kind of transmission, nevertheless, cannot be excluded, in particular in the case of intertidal and sub-tidal fish like herring. A similar hypothesis regarding exposure to animal and human sources could be put forward for marine mammals that may migrate along the coastline.

It remained to be established whether contact with edible marine fish colonized by *Blastocystis* sp. posed a risk of transmission to humans through their raw consumption and/or handling. As discussed above, among the 18 isolates subtyped in fish in the present study, 10 of them were either representative of potential new STs likely adapted to poikilothermic animals or of *Blastocystis* sp. lineages of as yet unknown origin and host specificity, all of them not yet identified in humans. Consequently, the risk of zoonotic transmission of these isolates appeared to be extremely limited, especially since some of them probably exhibit growth temperature that differs to those of isolates colonizing homeothermic animals [40,41,42]. In contrast, the other 8 isolates identified in fish belonged to ST2, ST7 or ST8 that have been found in the human population, with varying prevalence [17,18]. In particular, ST2 identified herein in whiting and mackerel represents one of the three most prevalent STs in humans with ST1 and ST3. Even if fishes are suggested not to be natural hosts of the mammalian ST2, ST7 and ST8, they can nonetheless be contaminated by these STs into the marine environment, thus acting as carriers of STs transmissible to humans. Globally, the potential for fish having a role as a major reservoir for zoonotic transmission of *Blastocystis* sp. infection seems limited but additional field investigations are recommended to improve our understanding of the role of edible marine fish as potential vector of this parasite and to evaluate the risk of foodborne transmission of *Blastocystis* sp. to human consumers.

Although several STs frequently found in humans [17,18] have been identified in marine mammals (ST2, ST3 and ST4), any direct contact between humans and these animals should be accidental and episodic. Therefore, the risk of zoonotic transmission is almost insignificant even though the faeces of marine mammals may transmit the parasite and may contribute to the contamination of the aquatic ecosystem.

## 4. Conclusions

To our knowledge, the present survey represents the first large-scale molecular epidemiological study conducted in the world focused on the prevalence and ST distribution of *Blastocystis* sp. in edible marine fish and marine mammals. Overall, the results of the study demonstrated that these animal groups were frequently colonized by *Blastocystis* sp. and could represent natural hosts of the parasite. Interestingly, new potential STs were identified in edible marine fish that could likely be considered as adapted to this animal group and more widely to poikilothermic organisms, including reptiles. More globally, our study confirmed the high genetic diversity observed within non-mammalian isolates with the current and future identification of numerous STs and the difficulty of establishing the origin and host specificity of the various *Blastocystis* sp. lineages. The further identification of additional isolates from fish will promote elucidating the genetic diversity of this parasite that probably still remains largely underestimated.

The influence of environmental factors such as seasonality or host factors such as fish species were shown to have an impact on *Blastocystis* sp. prevalence. Regarding the risk of zoonotic transmission of the parasite, it can reasonably be considered almost inexistent from marine mammals colonized by *Blastocystis* sp. due to episodic contact with humans. In the case of fishes, even though they are mostly infected with isolates not found in humans and probably adapted to poikilothermic animals, these animals are also transient hosts of STs with potential for human pathogenicity. Interestingly, some of these fishes, such as herring, that can be potential hosts for *Blastocystis* sp., are frequently eaten raw or after slight preparation and even without gutting [43]. However, the risk of zoonotic transmission can be considered rather limited in view of the low prevalence of fishes infected with mammalian STs. All these data provided substantial new insights into the understanding of the host range and transmission of *Blastocystis* sp. and stressed the importance of screening additional hosts in order to complete the epidemiology of this parasite. For instance, molecular data are lacking for marine mollusks [44], although these animals could represent an additional potential source of zoonotic transmission. In addition, molecular data remain extremely limited for amphibians whereas the prevalence of *Blastocystis* sp. has been shown to be very high in frogs and toads [42]. Since wildlife can potentially contribute to contamination of water, the identification of potential carriers of zoonotic *Blastocystis* sp. isolates is important for accurate risk assessment. In addition, in respect to public health, further studies are clearly required as the consumption of raw or slightly cooked fish is a novel eating habit.

## Figures and Tables

**Table 1:** Batches of fish analysed by real time PCR for the presence of *Blastocystis* sp.

Batch of Fish	Scientific Name	Common Name	Fishing Date	Fishing Area ^a^	Number of Individuals Per Batch (Number of Fishes Analysed Per Batch)	Number of *Blastocystis* sp.-Positive Fishes
CH-I	*Clupea harengus*	Herring	14/01/2014	27.VII.d Eastern English Channel	32 (10)	0
CH-II	*Clupea harengus*	Herring	02/12/2014	27.VII.d Eastern English Channel	30 (10)	4
CH-III	*Clupea harengus*	Herring	14/02/2015	27.VII.d Eastern English Channel	30 (10)	0
CH-IV	*Clupea harengus*	Herring	05/03/2015	27.VII.d Eastern English Channel	30 (10)	1
CH-V	*Clupea harengus*	Herring	08/12/2015	27.VII.d Eastern English Channel	30 (10)	0
CH-VI	*Clupea harengus*	Herring	08/12/2015	27.IV.c Southern North Sea	30 (10)	0
MM-I	*Merlangius merlangus*	Whiting	08/08/2014	27.IV.b Central North Sea	30 (15)	0
MM-II	*Merlangius merlangus*	Whiting	01/10/2014	27.VII.d Eastern English Channel	30 (20)	0
MM-III	*Merlangius merlangus*	Whiting	01/10/2014	27.VII.d Eastern English Channel	30 (20)	1
MM-IV	*Merlangius merlangus*	Whiting	27/01/2015	27.VII.d Eastern English Channel	30 (10)	1
MM-V	*Merlangius merlangus*	Whiting	01/04/2015	27.VII.d Eastern English Channel	30 (10)	0
MM-VI	*Merlangius merlangus*	Whiting	07/09/2015	27.VII.d Eastern English Channel	30 (10)	0
MM-VII	*Merlangius merlangus*	Whiting	07/09/2015	27.VII.d Eastern English Channel	30 (10)	0
PV-I	*Pollachius virens*	Saithe	16/05/2014	27.II.a Norwegian Sea	22 (10)	0
PV-II	*Pollachius virens*	Saithe	27/07/2014	27.IV.a Northern North Sea	30 (30)	0
PV-III	*Pollachius virens*	Saithe	02/10/2014	27.IV.a Northern North Sea	30 (10)	0
PV-IV	*Pollachius virens*	Saithe	27/11/2014	27.IV.a Northern North Sea	30 (10)	1
PV-V	*Pollachius virens*	Saithe	26/03/2015	27.IV.a Northern North Sea	30 (10)	1
PV-VI	*Pollachius virens*	Saithe	20/07/2015	27.IV.a Northern North Sea	30 (10)	0
SS-I	*Scomber scombrus*	Mackerel	07/08/2014	27.VII.d Eastern English Channel	30 (30)	0
SS-II	*Scomber scombrus*	Mackerel	17/09/2014	27.VII.d Eastern English Channel	20 (20)	2
SS-III	*Scomber scombrus*	Mackerel	01/10/2014	27.VII.d Eastern English Channel	30 (20)	1
SS-IV	*Scomber scombrus*	Mackerel	13/05/2015	27.VII.d Eastern English Channel	30 (10)	0
SS-V	*Scomber scombrus*	Mackerel	08/07/2015	27.VII.d Eastern English Channel	30 (10)	0
SS-VI	*Scomber scombrus*	Mackerel	07/09/2015	27.VII.d Eastern English Channel	30 (10)	0
SS-VII	*Scomber scombrus*	Mackerel	29/09/2015	27.VII.d Eastern English Channel	30 (10)	0

^a^ Divisions as determined by the International Council for the Exploration of the Sea ICES in the major fishing area 27 as shown in Figure 1.

**Table 2:** List of marine mammals stranded and tested by real time PCR for the presence of *Blastocystis* sp.

Individual	Scientific Name	Common Name	Date of Stranding	Place of Stranding (French Department) ^a^	Sex	Condition of Collected Sample ^b^	Sequence Positive Samples for *Blastocystis* sp.
CET-01	*Phocoena phocoena*	Common porpoise	25/01/2014	Saint-Etienne-au-Mont (62)	M	Fresh	0
CET-02	*Phocoena phocoena*	Common porpoise	06/03/2014	Boulogne-sur-Mer (62)	F	Putrefied	0
CET-03	*Phocoena phocoena*	Common porpoise	18/03/2014	Hardelot (62)	M	Putrefied	0
CET-04	*Phocoena phocoena*	Common porpoise	20/03/2014	Wimereux (62)	F	Fresh	0
CET-05	*Phocoena phocoena*	Common porpoise	27/03/2014	Boulogne-sur-Mer (62)	F	Putrefied	0
CET-06	*Phocoena phocoena*	Common porpoise	27/03/2014	Saint-Etienne-au-Mont (62)	M	Fresh	0
CET-07	*Phocoena phocoena*	Common porpoise	10/04/2014	Wimereux (62)	M	Fresh	0
CET-08	*Phocoena phocoena*	Common porpoise	21/08/2014	Ambleteuse (62)	M	Fresh	1
CET-09	*Phocoena phocoena*	Common porpoise	01/09/2014	Le Portel (62)	F	Very fresh	3
CET-10	*Phocoena phocoena*	Common porpoise	08/04/2014	Audresselles (62)	F	Fresh	0
CET-11	*Globicephala melas*	Long-finned pilot whale	02/11/2015	Calais (62)	M	Very fresh	0
CET-12	*Globicephala melas*	Long-finned pilot whale	02/11/2015	Calais (62)	F	Very fresh	0
CET-13	*Globicephala melas*	Long-finned pilot whale	02/11/2015	Calais (62)	M	Very fresh	0
CET-14	*Globicephala melas*	Long-finned pilot whale	02/11/2015	Calais (62)	M	Very fresh	0
CET-15	*Physeter macrocephalus*	Sperm whale	02/02/2016	Marck-en-Calaisis (62)	M	Putrefied	1
CET-16	*Phocoena phocoena*	Common porpoise	18/02/2016	Sangatte (62)	F	Fresh	0
CET-17	*Phocoena phocoena*	Common porpoise	29/02/2016	Audinghen (62)	M	Putrefied	0
CET-18	*Phocoena phocoena*	Common porpoise	28/02/2016	Audinghen (62)	M	Putrefied	0
CET-19	*Phocoena phocoena*	Common porpoise	29/02/2016	Audresselles (62)	F	Fresh	0
CET-20	*Phocoena phocoena*	Common porpoise	20/03/2016	Equihen-Plage (62)	F	Fresh	0
CET-21	*Phocoena phocoena*	Common porpoise	26/05/2016	Wimereux (62)	M	Putrefied	0
CET-22	*Phocoena phocoena*	Common porpoise	26/05/2016	Le Portel (62)	F	Putrefied	0
PIN-01	*Phoca vitulina*	Common seal	25/01/2014	Audinghen (62)	F	Fresh	0
PIN-02	*Phoca vitulina*	Common seal	06/03/2014	Tardinghen (62)	M	Fresh	0
PIN-03	*Phoca vitulina*	Common seal	22/12/2015	Camiers (62)	M	Fresh	0
PIN-04	*Phoca vitulina*	Common seal	18/01/2016	Wimereux (62)	M	Fresh	0
PIN-05	*Phoca vitulina*	Common seal	17/01/2016	Dunkerque (59)	F	Very fresh	0
PIN-06	*Phoca vitulina*	Common seal	11/03/2016	Saint-Etienne-au-Mont (62)	F	Fresh	2
PIN-07	*Phoca vitulina*	Common seal	18/03/2016	Oye-Plage (62)	M	Putrefied	0

^a^ As shown in Figure 1B. ^b^ Sample is considered very fresh when collected within 48 h after stranding.

**Table 3:** Sequence-positive samples for *Blastocystis* sp. in edible marine fish.

Batch and Individual	Fishing Area	Analysed Sample ^a^	Species	*Blastocystis* sp. ST best hit by BLAST	Sequence Identity with Best Hit ^b^
CH-II-1	Eastern English Channel	IC	Herring	ST8	100%
CH-II-1		SIE	Herring	ST10	92% ^c^
				(Untypable isolate ZLC7) ^d^	(100%)
CH-II-1		SGE	Herring	ST8	100%
CH-II-2	Eastern English Channel	IC	Herring	ST8	100%
CH-II-7	Eastern	IC	Herring	ST10	92% ^c^
	English Channel			(Untypable isolate ZLC7) ^d^	(100%)
CH-II-7		SIE	Herring	ST10	92% ^c^
				(Untypable isolate ZLC7) ^d^	(100%)
CH-II-7		SGE	Herring	ST8	100%
CH-II-8	Eastern	IC	Herring	ST10	92% ^c^
	English Channel			(Untypable isolate ZLC7) ^d^	(100%)
CH-II-8		SGE	Herring	ST7	98%
CH-IV-7	Eastern	IC	Herring	NMAST II ^e^	91% ^c^
	English Channel			(U-typable isolate ZLB27) ^d^	(100%)
MM-III-3	Eastern English Channel	IC	Whiting	ST2	100%
MM-IV-9	Eastern	IC	Whiting	NMAST VII ^e^	85% ^c^
	English Channel			(Untypable isolate LPO12) ^d^	(100%)
PV-IV-3	Northern	IC	Saithe	ST10	92% ^c^
	North Sea			(Untypable isolate ZLC7) ^d^	(100%)
PV-IV-3		SGE	Saithe	ST8	100%
PV-V-9	Northern	IC	Saithe	ST10	92% ^c^
	North Sea			(Untypable isolate ZLC7) ^d^	(100%)
SS-II-17	Eastern	IC	Mackerel	ST8	91% ^c^
	English Channel			(Untypable isolate LPA3) ^d^	(100%)
SS-II-46	Eastern English Channel	IC	Mackerel	ST2	100%
SS-III-1	Eastern	IC	Mackerel	ST10	92% ^c^
	English Channel			(Untypable isolate ZLC7) ^d^	(100%)

^a^ IC, intestinal content; SIE, scraping of the intestinal epithelium; SGE, scraping of the gastric epithelium. ^b^ When the level of sequence similarity reaches or exceeds 95% with a known ST, the amplicon is considered to belong to the considered ST. ^c^ SSU rDNA sequences included in the phylogenetic analysis. ^d^ Untypable isolates according to [16]. ^e^ NMAST, non-mammalian and avian ST [16].

**Table 4:** Sequence-positive samples for *Blastocystis* sp. in marine mammals.

Individual	Analysed Sample ^a^	Species	Cloning	*Blastocystis* sp. ST Best Hit by BLAST	Sequence Identity with Best Hit ^b^
CET-08	SIE	Common porpoise	N.d. ^f^	ST8	91% ^c^
				(Untypable isolate LPA3) ^d^	(100%)
CET-09	SIE	Common porpoise	Clone 1	ST4	99%
			Clone 2	NMAST VII ^e^	85% ^c^
				(Untypable isolate LPO12) ^d^	(100%)
			Clone 3	NMAST II ^e^	82% ^c^
				(Untypable isolate LPA10)	(99%)
CET-09	SGE-FU	Common porpoise	N.d.	ST2	99%
CET-09	SGE-FO	Common porpoise	N.d.	ST3	99%
CET-15	SIE	Sperm whale	N.d.	ST2	100%
PIN-06	IC	Common seal	N.d.	ST4	100%
PIN-06	SIE	Common seal	N.d.	ST4	100%

^a^ IC, intestinal content; SIE, scraping of the intestinal epithelium; SGE-FU, scraping of the gastric epithelium (fundus); SGE-FO, scraping of the gastric epithelium (forestomach). ^b^ When the level of sequence similarity reaches or exceeds 95% with a known ST, the amplicon is considered to belong to the considered ST. ^c^ SSU rDNA sequences included in the phylogenetic analysis. ^d^ Untypable isolates according to Reference [16]. ^e^ NMAST, non-mammalian and avian ST [16] ^f^ N.d., Not done.
